# Embedding a commitment to equitable global access into basic and early-phase translational research

**DOI:** 10.1017/cts.2024.691

**Published:** 2025-02-25

**Authors:** Barry S. Coller

**Affiliations:** Rockefeller University, New York, NY, USA

**Keywords:** Technology transfer, patent, license, manufacturing, health equity

## Abstract

The COVID-19 pandemic laid bare the inequities in U.S. healthcare in ways that captured public attention and reinforced the need to view all of healthcare through an equity lens. It also exposed global inequities in access to healthcare technologies. At Rockefeller University, we participate in the entire spectrum of translational research, but our focus is in the areas of basic research and new methods to prevent, diagnose, and treat disease, extending to proof of concept preclinical and Phase 1 studies. Since we believe that all phases of translational research should have an equity lens, we have instituted an initiative to encourage thought and planning about global equitable access to discoveries made by our trainee Clinical Scholars and faculty, even at the earliest phases of basic research. Assuring global equitable access to new technologies requires addressing at least 3 different aspects of new technology: 1. Patenting and licensing, 2. Manufacturing, and 3. Dissemination and implementation in low- and middle-income countries. In this review, I focus on patenting and licensing and offer ten questions for inventors to consider in discussing licensing their technologies with technology transfer officers to maximize equitable global access to the technologies they create.

## Introduction

**Figure 1.**
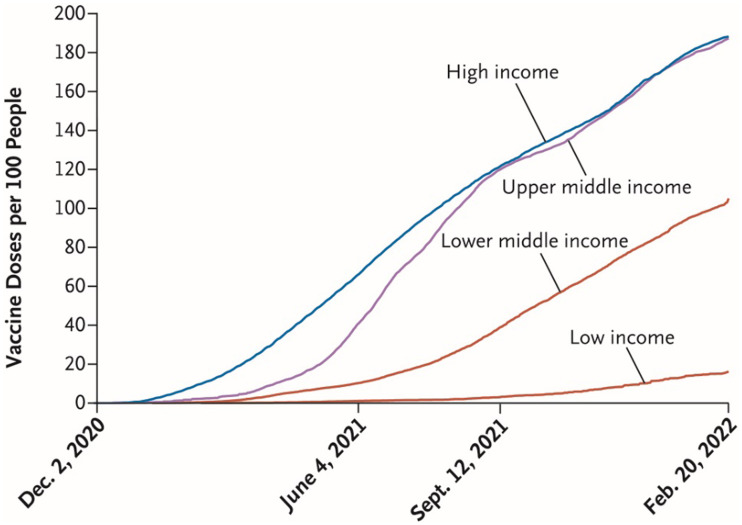
COVID-19 vaccine doses administered in countries categorized by income level, December 2, 2020, to February 20, 2022. Income categories are those defined by the World Bank. Data are from Our World in Data (https://ourworldindata.org/covid-vaccinations). Reprinted with permission from Hunter et al., Addressing vaccine inequity — COVID-19 vaccines as a global public good, N Engl J Med 2022; 386: 1176–1179.

The COVID-19 pandemic laid bare the inequities in US healthcare in graphic ways that captured public attention and reinforced the need to view all of healthcare through an equity lens. It also exposed the global inequities in access to healthcare technologies crucial for responding to the pandemic and protecting the public. In particular, access to vaccines differed dramatically among most high-, middle-, and low-income countries (Figure 1) [1–4].

**Figure 2.**
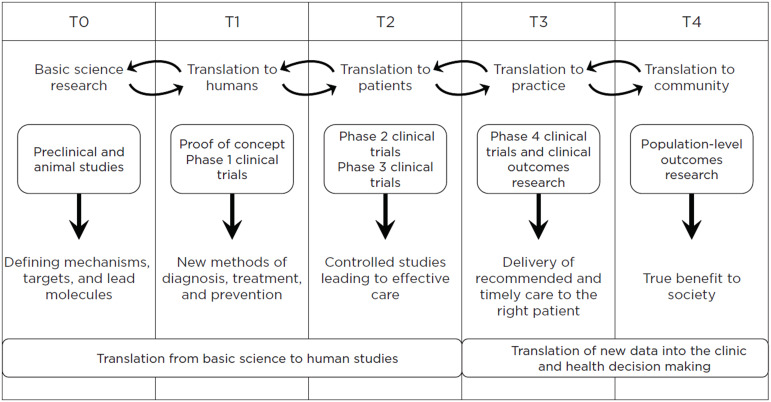
Taxonomy of translational research. T0 research: basic biomedical research, including preclinical and animal studies, not including interventions with human subjects; T1 research: translation to humans, including proof of concept studies, Phase 1 clinical trials, and focus on new methods of diagnosis, treatment, and prevention in highly-controlled settings; T2 research: translation to patients, including Phase 2 and 3 clinical trials, and controlled studies leading to clinical application and evidence-based guidelines; T3 research: translation to practice, including comparative effectiveness research, post-marketing studies, clinical outcomes research, as well as health services, and dissemination and implementation research; and T4 research: translation to communities, including population level outcomes research, monitoring of morbidity, mortality, benefits, and risks, and impacts of policy and change. Figure reprinted with permission from the Institute of Medicine. *The CTSA program at NIH: Opportunities for Advancing Clinical and Translational Research*. Washington, DC: the National Academies Press National Academy of Sciences, Engineering, and Medicine. 2013. https://doi.org/10.17226/18323 and text adapted from University of Wisconsin Institute for Clinical and Translational Research (https://cancer.wisc.edu/research/otrs/process/).

The NIH Clinical and Translational Science Award (CTSA) program has made research on identifying and addressing health disparities a high priority [5–17]. The vast majority of this research has focused on equity in the diagnosis and treatment of disease and public health policy, with a major focus on engaging communities throughout the clinical research process. These activities are primarily in the T3 (translation of research findings into clinical practice) and T4 (translation of research findings into real-world populations) end of the spectrum of translational research as defined by the Institute of Medicine 2013 report on the CTSA program (Figure 2) [18]. At Rockefeller University, we participate in the entire spectrum of translational research; however, our focus is in the T0 (basic science research) and T1 (translation to humans) areas of basic research and studying new methods to prevent, diagnose, and treat disease, extending to proof of concept preclinical and Phase 1 studies. Since we believe that all translational research should have an equity lens, we have instituted an initiative to encourage thought and planning about global equitable access to discoveries made by the junior translational scientists in our Clinical Scholars program, supported in part by our CTSA KL2 training program, and faculty, even at the earliest phases of basic research. As a first step along this path, we devote several tutorials to encouraging the Clinical Scholars to consider the answers to two key questions: 1) Assuming that your basic research is successful and you are able to develop a new drug, biologic, biomarker, diagnostic, or device, what do you anticipate will be the major obstacle(s) to equitable global dissemination? 2) What can you do now to minimize those obstacles by the design of your project or in future licensing, manufacturing, sales, and distribution?

The answers to these questions need to address at least three aspects of the technology: 1. Patenting and licensing; 2. Manufacturing; and 3. Dissemination and implementation in low- and middle-income countries (LMICs). This paper will focus on patenting and licensing and just touch on manufacturing, leaving dissemination and implementation for future analysis. It is important to emphasize, however, that challenges in infrastructure and resources to support clinical studies and delivery of new technologies in LMICs are crucial in achieving equitable global access, and these challenges need to be addressed by governmental agencies and nongovernmental organizations, ideally working with the academic community [19–21].

## Patenting and licensing

**Constitutional Authorization.** Article I, Section 8, Clause 8 of the United States Constitution, grants Congress the power “To promote the progress of science and useful arts, by securing for limited times to authors and inventors the exclusive right to their respective writings and discoveries.” The goal was to promote public good, not private gain. As products of the Enlightenment, the founders recognized the importance of scientific progress and judged that the best way to speed progress was to encourage inventors to fully disclose their invention – a requirement for obtaining a patent – so that others could improve on the patented invention, rather than inventors keeping their inventions as “trade secrets.” Moreover, the founders recognized that to finance the development of an invention to the point where it would be useful to the public, it would likely be necessary to obtain capital from investors who expected a return on their investment. If there were no patent protection, others could wait until development was completed and only bear the manufacturing costs rather than both the development and manufacturing costs. Thus, although the founders recognized the dangers of monopolies, they sought to balance the incentive to patent against the public’s interest in obtaining products at the lowest cost by limiting the time of exclusivity. The patent system has evolved dramatically since the Constitution was written, and there are serious concerns about whether it currently is optimal for the modern technological age [22]. In addition, biotechnology, pharmaceutical, and medical device patents represent only a fraction of total patents issued, and so the patent system must balance the impact of specific policies and procedures across a wide range of industries.

For most academic investigators, the decision as to whether to patent an invention is made by the university’s technology transfer office or its equivalent. Universities differ in the criteria they use to make that decision, and in some cases, university and National Institutes of Health (NIH) policies may allow an investigator to privately apply for a patent if the university chooses to not apply. A key factor in a technology transfer office’s decision to file a patent application is whether the time-limited monopoly that a patent enables will likely incentivize a company to license the technology and invest in its further development. Such partnership is crucial for the development of technologies that require capital beyond that available in academic organizations. Without a patent, opportunities for licensing diminish greatly, which may preclude obtaining the necessary funds and partnerships to fully develop an invention into a useful product. Obtaining a patent in high-income countries is most important for economic success, and so the decisions as to whether to also apply for patents in LMICs, to receive royalties for licensing the invention to companies in LMICs, or to enforce a patent in LMICs can potentially be separated in the pursuit of global equitable access to novel agents. In fact, many of these factors may come into play during the licensing process, which is discussed below, and there are examples of universities using their patent rights to encourage pharmaceutical companies to maximize global access [23].

**The Bayh-Dole Act** [24]. The 1980 Patent and Trademark Law Amendments Act of 1980, commonly called the Bayh-Dole Act in recognition of the two Senators who sponsored it, gave the privilege of licensing patented technology developed with federal funding to the institution receiving the federal funding. This was in response to evidence obtained at that time showing that technology invented and developed with federal funds were rarely patented or converted into products by private industry. A General Accounting Office report noted that at the time Bayh-Dole was enacted, “fewer than 5 percent of the 28,000 patents being held by federal agencies had been licensed, compared with 25 percent to 30 percent of the small number of federal patents for which the government had allowed companies to retain title to the invention [25].” The Act encouraged universities to patent and license new technologies, and it encouraged academic scientists to develop such technologies by requiring that universities share some of the proceeds from licenses with the inventor(s). It also provided for the government to retain “march-in” rights for the product or technology that was patented if “action is necessary to alleviate health or safety needs that are not reasonably satisfied by the contractor, assignee, or their licensees [26].” The government could then license to other companies. There has been controversy as to whether the high price of a drug would qualify as a justification for exercising “march-in” rights, and there are legal and practical complexities that have discouraged the exercise of these rights. Despite several petitions to the government to exercise these rights on specific products, the government has not yet done so on any product [26].

While the Act has been criticized for encouraging universities to focus on commercialization at the expense of their academic mission, it unequivocally unleashed a major wave of discoverythat has resulted in many important products, including CRISPR-Cas9 for human gene editing and the Google search engine. According to a yearly survey conducted by the Association of University Technology Managers (AUTM), in 2020, 184 US academic institutions applied for 17,738 patents and received about 8,706 [27]. In the same year, 1,117 new companies were created to commercialize university inventions. AUTM’s data from 1996 to 2020 indicate that licensed academic technologies contributed between $333 billion-$1 trillion to the gross domestic product and produced between 2.4 and 6.5-million person-years of employment [28].

**Critiques of University Patenting and Licensing, Responses to the Critiques, and Suggested Alternatives.** From its inception, the Bayh-Dole Act engendered criticism about the impact of commercialization of university technology on the educational and public interest missions of universities. Critics pointed to a number of examples of how exclusive university licenses restricted access to important drugs in LMICs, stimulating public concern [23,24]. In response, States initiated investigations, student and faculty organized protests, and the student-led Universities Allied for Essential Medicines (UAEM) issued a 2006 manifesto that called for institutions to “promote equal access to university research” by focusing less on profit and more on human welfare [29,30]. This led to representatives from thirteen research-intensive institutions developing a set of guidelines the next year calling on academic institutions to prioritize their educational and public missions rather than their economic interests in licensing technology to the private sector [24]. The resultant document, titled *In the Public Interest: Nine Points to Consider in Licensing University Technology (9P)* [31], identified key provisions for principled licensing, focusing on both academic freedom and equitable global access (Table 1). Since then, 118 universities and related entities have signed on to the document, and it has been endorsed by the National Research Council and the Association of American Universities [24]. It thus enjoys widespread support.

**Table 1.** Provisions of *In the Public Interest: Nine Points to Consider in Licensing University Technology* [31]

Point 1. Universities should reserve the right to practice licensed inventions and to allow other nonprofit and governmental organizations to do so. This is to ensure that the invention is freely available for educational purposes and further research at the licensing institution and other nonprofit and governmental institutions.
Point 2. Exclusive licenses should be structured in a manner that encourages technology development and use. Exclusive licenses may be necessary in order to obtain a commitment of substantial resources to develop a product, especially drugs, vaccines, and devices, but they raise concerns that should be addressed in the license agreement. 1. The licensee may not commit the resources necessary to develop the technology for the anticipated application or may abandon development for financial or other reasons unrelated to the value of the technology for improving human health. Thus, “it is important that licensees commit to diligently develop the technology,” including providing adequate resources and meeting agreed-upon time-limited performance milestone. Failure to do either should be grounds for terminating the license so that the university can license to others. 2. The public health needs may outstrip the ability of the licensee to meet the needs. To address this, the license should “require exclusive licensees to grant sublicenses (or direct licenses) to third parties to address unmet market or public health needs (“mandatory sublicensing”).” 3. The technology may have unanticipated utility outside of the core commercial interest of the licensee, and under those circumstances it is in the public’s interest that others are able to develop the technology for those additional applications. Thus, it may be desirable to limit exclusivity under those circumstances.
Point 3. Strive to minimize the licensing of “future improvements.” Licensees often want to make sure that if the technology is improved by the inventor as a result of their future research, that they have access to that improved technology. This is commonly achieved by licensing such future technology in a particular “field.” The problem is that “the obligation of such future inventions may effectively enslave a faculty member’s research program to the company, thereby exerting a chilling effect on their ability to receive corporate and other research funding and to engage in productive collaborations with scientists employed by companies other than the licensee – perhaps even to collaborate with other academic scientists.” In some extreme cases, committing to future improvements may also unwittingly have an impact on other investigators at the same university! The 9P recommends that “licensed rights should be limited to existing patent applications and patents, and only to those claims in any continuing patent applications that are (i) fully supported by information in an identified, existing patent application or patent and (ii) entitled to the priority date of that application or patent.”
Point 4. Universities should anticipate and help to manage technology transfer-related conflicts of interest. It is important that university technology transfer officials and university officials responsible for the oversight of institutional conflict-of-interests have open communication to ensure that licensing activities do not create inappropriate conflicts of interest.
Point 5. Ensure broad access to research tools. 9P cites the NIH Guidelines on Research Tools [86,87], along with principles established by charitable foundations and expectations of all academic institutions to make research tools “as broadly available as possible.” Scientific journals have also strengthened their requirements about making unique resources available so that others can verify the authors’ results and build on their discoveries. Thus, the licensing of research tools requires considerable thought and the balancing of the need to incorporate sufficient economic incentive to achieve further development and distribution of important reagents, kits, or devices for research, diagnostics or other uses, while not creating barriers to access. One suggestion made by P9 is to consider limiting exclusivity to the sale, but not the use of such products or services. This allows the university the right to license non-exclusively to others the right to use the patented technology using either the products purchased from the exclusive licensee or materials made in-house. It tried to address what was a major concern when Myriad Genetics exploited its position as the only authorized provider of BRCA1/BRCA2 diagnostic testing in the USA to prevent further research and the use of the technique in multi-gene analysis [24,88]. If at all possible, research tools should be licensed non-exclusively. Some scholars have even questioned the rationale for being able to patent research tools [23].
Point 6. Enforcement action should be carefully considered. Universities should try to resolve licensing and patenting issues short of litigation, but if litigation is the only resort, the university needs to present the rationale not only in court but also to the public.
Point 7. Be mindful of export regulations. Licenses must comply with federal export control laws and licensing “proprietary or confidential” information may affect the “fundamental research exclusion” enjoyed by most university research [24].
Point 8. Be mindful of the implications of working with patent aggregators. Since most university patents are never licensed, it is tempting for universities to license patents that have not attracted commercial interest by a company in the field of the patent to license such patents to patent aggregators. Some aggregators play an important role in the public interest by assembling a group of patents that represent both foundational patents and later improvements for the purposes of facilitating development of new technologies. Other aggregators (“patent trolls”), however, assemble large numbers of patents and then use them to frighten companies into licensing them to avoid potential patent infringement lawsuits.
Point 9. Consider including provisions that address unmet needs, such as those of neglected patient populations or geographic areas, giving particular attention to improved therapeutics, diagnostics, and agricultural technologies for the developing world. 9P recognized that universities have a responsibility to “share the fruits of what we learn globally, at sustainable and affordable prices, for the benefit of the world’s poor,” and that “[u]niversities should strive to construct licensing arrangements in ways that ensure that these underprivileged populations have low- or no-cost access to adequate quantities of these medical innovations.” 9P did not provide details on how best to achieve these goals, acknowledging the complexity of the problem given the lack of alignment of economic and socially responsible goals.

The 9P document was followed by a 2009 *Statement of Principles and Strategies for the Equitable Dissemination of Medical Technologies* by AUTM and a group of academic institutions to provide “a more concrete statement of goals as well as licensing practices [32].” Among its provisions was a commitment to “to contribute to the health and well-being of populations throughout the developing world” by reducing intellectual property barriers through: 1. Not patenting in developing countries. 2. Filing and abandoning patents. 3. Patenting in developing countries when there are potential benefits, as for example, when there is a pharmaceutical manufacturing capacity in the country, or the patent would provide leverage to obtain concessions that would increase access. When patents are obtained, institutions should consider: 1. Offering licensing terms that incentivize making the technology available in developing countries; 2. Including “march-in” rights, mandatory sublicenses, or non-assert provisions; 3. Obligating licensees to meet milestones or face license reduction, conversion to non-exclusivity, or termination; and 4. Requiring tiered, subsidized, at-cost, or no-cost pricing. The 2009 statement also called for partnerships with private companies, government, and not-for profit organizations, as well as the development of “meaningful metrics” to assess the success of the initiative.

In 2023, Jorge Contreras conducted a study of the impact of the 9P on university licensing using primarily data reported by companies to the Securities and Exchange Commission [24]. As a result, the data are skewed toward companies in which the license was considered “material” to the companies’ economic status. Based on 220 university technology licenses completed before and after the publication of 9P, he concluded that 9P increased the availability of the licensed technologies for education and nonprofit research, but there were few changes related to public health and global equitable access. He called for encouraging academic faculty, senior administrators, students, alumni, and other institutional stakeholders to participate in the process of developing university technology transfer policy, rather than leaving it to the technology transfer office, trustees, and the most senior institutional leadership. He also noted that the ranking metrics used by AUTM focus on commercial accomplishments, such as licensing income and startup formation rather than public benefit, and 39 of 172 universities reported in the 2017 AUTM salary survey using such metrics for incentive compensation of Technology Transfer Office personnel. AUTM does, however, also highlight non-monetary metrics, including new commercial products launched (a measure of impact), startup companies formed and those formed in the institution’s home state (measures of economic development), and number of disclosures and licenses (a measure of faculty engagement in commercial innovation).

The UAEM is student-created advocacy group that seeks to improve global health by influencing university policies nationwide. In 2006, it released its *Philadelphia Consensus Statement: On University Policies for Health-Related Innovations* [30]; in 2010, it released its *Global Access Licensing Framework* [33]; and in 2021 it released its *Equitable Technology Access Framework (ETAF)* [34]. The ETAF has three goals: 1. To improve global equitable access to health technologies, 2. Promote further development of health technologies, and 3. Improve transparency of health technology transfer. These goals are supported by nine principles for technology transfer offices to incorporate into their operations: 1. Social responsibility to the public, 2. Limit monopolies, 3. Retain IP rights, 4. Include step-in rights, 5. Include reach-through clauses, 6. Limit data and market exclusivities, 7. Commit to full sharing of all data and research findings, 8. Ensure full transparency of all public funding sources and amounts, and 9. Implement robust accountability and transparency mechanisms. In 2024, UAEM produced a white paper that described the history of the organization and the evolution of its advocacy efforts. It compares its ETAF to the 9P and finds the latter wanting because the 9P does “not require accountability, transparency, or limitations on the creation of predatory monopolies.” UAEM also notes that the 9P and the 2009 *Statement of Principles* do not address access to medications for poor people in high income countries. UAEM issued a report in 2013 that evaluated 54 major North American universities based on their investment in global-health innovations, especially neglected diseases, policies supporting global equitable access, and empowering and educating future global health leaders [3]. The University of British Columbia was the only institution to receive an overall grade of A, and Vanderbilt, Harvard, and Northwestern were the only universities to receive an A in more than one category. Institutions that had high innovations scores tended to have low scores for policies to ensure equitable global access, and vice versa [3]. In fact, the University of British Columbia does its own assessment of its licenses based on their academic and social benefit and their economic, financial, and political impact [3]. The steady increase in noncommunicable diseases as public health threats in LMICs raises the question as to whether a focus on innovation for neglected diseases in the score card is still appropriate [3]. UAEM issued another report in 2020, adding to the original criteria analyses of transparency in clinical trial results and academic medical publications, and sharing the intellectual property, knowledge, and data from their COVID-19 research in ways that ensured equitable global access [35].

In the philanthropic sphere, the Bill and Melinda Gates Foundation developed a Global Access policy in 2003 to ensure that the products of foundation-funded projects benefit the foundation’s beneficiaries, which they define as “the people most in need living in developing countries and within the USA.” The policy requires that products are disseminated promptly and broadly, and that they are “made available and accessible at an affordable price.” The grant recipient may also be required to create a “Global Access Strategy” that outlines plans for managing intellectual property and the associated rights, including manufacturing and distribution, and may require a Humanitarian License to achieve these goals.

Spurred by a faculty member’s invention to improve the diagnosis of dengue fever [36], the University of California at Berkeley developed its Socially Responsible Licensing Program in 2003, which has the following goals: 1. Promote widespread availability of healthcare and technologies in the developing world, 2. Maximize societal impact and public benefit of technologies developed at Berkeley, 3. Share revenue and/or other benefits with those who collaborate with Berkeley researchers, 4. Give proper attribution to a resource/material provider or collaborator, and 5. Stimulate additional investment by others to achieve these goals [37]. One innovation of this program was to change the metrics for assessing the success of their technology transfer program. For example, while granting a royalty-free license may decrease the revenue collected by the licensing office, the office will get credit for net funding, philanthropic gifts, relationships, and campus recognition produced by the license [36].

Similarly, in 2020, the University of California, Los Angeles (UCLA), began requiring licensees to provide and implement an “Affordable Access Plan” that includes a list of LMICs in which the licensee does not intend to commercialize the product, as well as plans (including strategies and timelines) to support affordable access in LMICs and non-commercialized territories. UCLA’s adoption of the Affordable Access Plan approach grew out of its discussions with the Medicines Patent Pool (MPP), a nonprofit organization developed by Unitaid, an international organization that provides financial support for the development of innovations to prevent, diagnose, and treat HIV/AIDS, tuberculosis, viral hepatitis, and malaria [38]. MPP is a hosted partnership with the World Health Organization that uses voluntary licensing and patent pooling, along with collaborations with organizations, industry, patient groups, and governments to prioritize licensing to LMICs [39]. Its creation was spurred by the World Trade Organization’s 1995 agreement on Trade-Related Aspects of Intellectual Property Rights, which required all countries to offer patents on pharmaceuticals. Among its tactics, MPP provides licenses to generic pharmaceutical companies based anywhere in the world to manufacture drugs [40,41]. MPP has been endorsed by the G7, G20, and the United Nations. As of 2023, MPP has signed 34 licenses, established partnerships with 58 manufacturers, facilitated access to 30 billion doses of treatments, and achieved savings of $1.2 billion in health care costs. Among its new goals is expanding its efforts to non-communicable diseases and maternal health. MPP also encourages transparency through hosting publicly available databases on patents on essential medicines (MedsPath), COVID vaccines (VaxPal), and long-acting therapeutics (LAPaL).

Although pharmaceutical companies can license directly to generic companies themselves, MPP licenses: 1. Generally enable more generic companies to produce drugs, thus increasing competition and lowering costs; 2. Usually involve larger territories; 3. Frequently allow exports outside of the licensed territory; and 4. Are made publicly available on the MPP website [40]. Two COVID-19 drugs, molnupiravir and nirmatrelvir were licensed to MPP by major pharmaceutical companies before their global launch and others have been licensed while still in development. The MPP model works better for small molecules than for biologics or vaccines because the latter two require more transfer of “know-how,” that is, technical details of the manufacturing process. Even with these limitations, universities could require that licensees make reasonable efforts to engage in future licensing to MPP for distribution in LMICS in lieu of direct licensing to generic companies. Similarly, nonprofit funders could adopt the same requirements and governments could incentivize pharmaceutical companies to license to MPP via a series of rewards.

Academic institutions can themselves craft licenses to achieve the same goals. For example, Rockefeller University recently completed a licensing transaction with an organization located in an LMIC for a therapeutic to treat a disease with unmet need. The focus of the license was on “dissemination” rather than “sales” of the technology; making the treatment available at-cost resulted in a royalty-free license. It is heartening that both public and private institutions are leading in developing creative approaches because the funders of each may have somewhat different expectations about their technology transfer programs, with public institutions often seen as drivers of local economic development and private institutions variably dependent on royalty income.

The “Open Science” movement has challenged the entire model of academic technology transfer, citing many negative consequences, including delays in moving technology forward because of the need for protracted negotiations with universities, diversion of student and faculty attention from scientific discovery to personal financial gain, overly aggressive university demands and expectations, licensing of inventions too early in their development, and wasting university resources because few technology transfer offices bring in enough money to cover their costs [42–44]. The alternative is the development of collaborations among academic institutions, industry, and philanthropic institutions to speed technology development free from intellectual property constraints. One example of this model is the Canadian Structural Genomics Consortium (SGC), which is funded by major pharmaceutical companies and includes partners from government and philanthropy who collaborate with academic investigators on scientific research without retaining intellectual property rights. The SGC thus represents an open science model in precompetitive structural and chemical biology, which enables “companies and research institutions to share costs and risks associated with the large-scale efforts required to elucidate new therapeutic opportunities from underexplored areas of the human genome [45].” It entered into a partnership with the Ontario Institute for Cancer Research to explore a potential novel target for treating malignancies. Based on data obtained by international partners, the target became validated. At that point, the institute developed a drug for leukemia which it patented and licensed to Celgene for a $40 million upfront payment and the potential to receive as much as $1 billion. M4K, an Open Science company focused on developing drugs for rare pediatric disorders, is committed to publicly sharing all of its data and does not file patents. It is owned by the Agora Open Trust, a Canadian charity committed to Open Science principles [43,46]. When companies owned by the Agora Open Trust develop a technology to the point where it is appropriate to partner with a company, instead of licensing a patent, the company gets exclusive rights to the data packages submitted to regulators [47]. The licensee must ensure that the technology is equitably accessible, but how that is achieved is not specified.

An alternative Open Science route has been championed by the Center for Vaccine Development, Texas Children’s Hospital, Baylor College of Medicine, which is Co-Directed by Drs. Peter Hotez and Maria Elen Bottazzi. The Center is committed to performing research to produce vaccines for neglected diseases in LIMCs. It tries to avoid dependance on multinational pharmaceutical companies altogether because, as shown by the COVID-19 experience, they prioritize supplying North American and European countries, charge high prices, and do not have the capacity to supply the needs of LMICs. In response, their group produced a recombinant protein vaccine made by microbial fermentation in yeast and then transferred the prototype for production by vaccine producers in India (Biological E; Corbevax; available at ∼$3.00/dose) and Indonesia (BioFarma; IndoVac) [48–50]. They also published papers on each step in the vaccine development process and made them available through open access. Baylor College of Medicine offered the manufacturers nonexclusive licenses without patent protection. In addition, since vaccine production is more complex than manufacturing small molecules, they also committed to sharing their know-how and their quality control and quality assessment documents. In addition, they trained scientists from abroad on vaccine development, production, and quality control. Approximately 100 million doses of the vaccine have been administered in these two countries; however, regulatory hurdles related to the need for approval by stringent regulatory authorities have limited adoption by other LIMCs [48]. The approach taken by the Baylor group has the added advantage of supporting the development of infrastructure, skills, and know-how in LMICs, thus building the future capacity to address key health problems of specific importance to LMICs.

In a similar vein, one need not view academic licensing under Bayh-Dole and Open Science as polar opposites. For example, in the 1980s, long before the promulgation of the 9P, the author was able to obtain agreement from the technology transfer officer at the Research Foundation of the State University of New York and the licensing company Centocor to allow him to share the monoclonal antibody that reacts with the platelet integrin receptor αIIbβ3 – the parent antibody of the drug abciximab – with academic investigators for research purposes without charge and without obligations regarding future authorship or confidential sharing of the research results. Sharing the antibody yielded some of the benefits of an Open Science collaboration as the hundreds of investigators who received free antibody began publishing data in the scientific literature. The results of their studies were very important in the development of abciximab, demonstrating the benefits of such a policy [51,52]. The first point in the 9P recommends that licenses include these “reservation of rights,” and Contreras found that this recommendation had the greatest uptake, with reservation of rights for research purposes for all nonprofit organizations in the academic licenses reviewed increasing from 43% before to 73% after 9P was published [24]. In addition, all of the studies carried out by the author, including those conducted in collaboration with Centocor scientists, were published in the scientific literature [53–77]. Similarly, the technology transfer officer at Rockefeller University and the licensing company CeleCor allowed a similar provision in the license for the compound RUC-4 (zalunfiban), which also targets αIIbβ3, and is currently under development [78–81]. Academic investigators have already independently published data confirming that zalunfiban does not induce a conformational change in the receptor, a key distinguishing feature of the compound [82]. The Open Science movement has thus challenged many of the assumptions of the dominant academic model of drug and vaccine development, and so beyond its successes to-date, it is important to assess whether its principles are generalizable to a broad range of drug and vaccine development projects.

## Manufacturing

The ability to manufacture drugs and biologics at low cost and in large numbers is crucial for equitable global access. There are, however, major challenges in creating a sustainable economic model for manufacturers in LMICs because of uncertain demand, the need for expensive equipment for large-scale manufacturing, and the need to comply with exacting regulatory standards. Fortunately, there is increasing capacity for vaccine manufacturing in LMICs and many manufacturers would like to expand their operations [83]. It is also relatively straightforward to manufacture small molecule drugs, but vaccines and other biologics often require transfer of advanced know-how, and some manufacturing processes are complex, such as those that are used to produce mRNA vaccines in lipid nanoparticles. That is one reason that the Center for Vaccine Development, Texas Children’s Hospital, chose to make its vaccine by a yeast protein expression technology that is well known, and thus easily adopted in manufacturing facilities in LIMCs. Local production also provides jobs and enhances the skills of the local population. The need for stringent regulatory authority approval (e.g., by the US Food and Drug Administration or the European Medicines Agency) in order to sell products outside of the country in which it is manufactured – even if the product has national approval – has, however, been a major stumbling block and advocates for global access are trying to address this challenge in a number of different ways [48,83]. Multinational pharmaceutical companies are also establishing manufacturing facilities in LMICs, but it is not clear how their presence will affect the ecosystem.

Contreras and Shadlen recently compared the vaccine development approach taken by the Center for Vaccine Development, Texas Children’s Hospital to that taken by Oxford University [1]. The Oxford group initially indicated that it would offer royalty-free, nonexclusive licenses to COVID-19 technologies, but subsequently licensed its vaccine exclusively to AstraZeneca with a royalty return to Oxford [1]. As a result, the Oxford vaccine was produced by a network of manufacturers that produced 3 billion doses, most of which were distributed in LMICs at “low cost,” compared to the 100 million doses of the Center for Vaccine Development’s vaccine. They highlighted advantages of the Oxford approach, including the faster regulatory approval of the Oxford vaccine, the transfer of know-how in addition to patent rights by Oxford, greater access to capital, and knowledge about selecting partners in global manufacturing, including a major manufacturer in India. They noted that the Oxford technology was much more complex and thus needing more transfer of know-how, but did not indicate that Center for Vaccine Development specifically selected a vaccine production method that would be easier to adopt in LMICs. They did not provide data on the pricing of the Oxford vaccine, admitting that the pricing was “opaque and inconsistent.” Moreover, they did not acknowledge that regulatory structures, which are amenable to modification, limited other countries from receiving the Center for Vaccine Development’s vaccine made in India and Indonesia, which likely resulted in many fewer doses of the vaccine being produced by the two countries. Their analysis highlights the complexity of maximizing equitable global access and indicates what governmental and nonprofit organizations need to do to match the benefits that AstraZeneca had in achieving its success in manufacturing at the enormous scale required for global distribution.

## Conclusions

Basic scientists and early-phase translational investigators in universities focus their attention on scientific discovery and the development of technologies that address important human health needs. Since those discoveries and technologies are the earliest steps in clinical translation, it is important for them to consider the health equity issues that may emerge downstream from their discoveries. It is vital, therefore, for academic investigators to work collaboratively with their university technology transfer office to optimize global equitable access to their technology. This requires recognition of the complexity of patenting and licensing and appreciating the expertise of the technology transfer personnel. That is why we have initiated our educational program for our KL2 Clinical Scholars and plan to assess the impact of the program on their future translational activities. An increasing number of investigators are participating in courses on entrepreneurship, including those sponsored by the National Science Foundation’s Innovation Corps (I-Corps), where they can learn some of the fundamentals of product development and commercialization [84,85]. Table 2 contains a series of questions that investigators may want to consider as they work with their technology transfer colleagues to craft the best strategy for licensing their technologies, realizing that the details of patenting and licensing will have a major impact on the manufacturing, dissemination, and implementation of their technology, which will, in turn, determine whether there is equitable global access to the technology and whether the technology will ultimately help reduce health disparities around the world.

**Table 2.** Questions for inventors to consider in discussing licensing their technologies with technology transfer officers to maximize equitable global access to the technologies they created

1. Will the license allow the university to provide the technology to academic investigators for research purposes without costs exceeding those incurred by the university in preparing and sending the materials?
2. What are the pros and cons of patenting the technology with regard to equitable global access?
3. What are the pros and cons of exclusive versus non-exclusive licensing of the technology?
4. If the decision is made to apply for patent protection, what will determine the choice of countries in which to seek a patent(s)?
5. Will the technology be non-exclusively licensed, with no or limited royalties, to companies in low- and middle-income countries (LMICs) in which no patent applications were submitted?
6. Will the university be willing to include financial incentives in exchange for reduced or at-cost pricing in LMICs?
7. Will the university include license terms that encourage a licensee to pursue actions to ensure that people in the USA who lack the resources to purchase or gain access to the technology can still obtain access to it?
8. Will the university require that the licensee commit to providing the funds necessary to develop the technology and that failure to meet agreed-upon milestones in the development process will result in the license being returned to the university?
9. Where applicable, will the university strive to include in licenses terms that relate to downstream non-exclusive licensing to the Medicine Patent Pool (MPP) to enable the MPP to grant licenses to generic manufacturers in LMICs?
10. Will the university require that a licensee provide an “Affordable Access Plan” like that required by UCLA?
